# Gradient Index Devices for the Full Control of Elastic Waves in Plates

**DOI:** 10.1038/srep24437

**Published:** 2016-04-14

**Authors:** Yabin Jin, Daniel Torrent, Yan Pennec, Yongdong Pan, Bahram Djafari-Rouhani

**Affiliations:** 1 School of Aerospace Engineering and Applied Mechanics, Tongji University, 100 Zhangwu Road, 200092, Shanghai, China; 2 Institut d’Electronique, de Micro électronique et de Nanotechnologie, UMR CNRS 8520, Université de Lille 1, 59655 Villeneuve d’Ascq, France; 3 Université de Bordeaux, Centre de Recherche Paul Pascal, UPR 8641, 115 Avenue Schweitzer, 33600 Pessac, France

## Abstract

In this work, we present a method for the design of gradient index devices for elastic waves in plates. The method allows the design of devices to control the three fundamental modes, despite the fact that their dispersion relation is managed by different elastic constants. It is shown that by means of complex graded phononic crystals and thickness variations it is possible to independently design the three refractive indexes of these waves, allowing therefore their simultaneous control. The effective medium theory required for this purpose is presented, and the method is applied to the design of the Luneburg and Maxwell lenses as well as to the design of a flat gradient index lens. Finally, numerical simulations are used to demonstrate the performance of the method in a broadband frequency region.

The control of the propagating characteristics of mechanical (acoustic and elastic) waves in linear media is a challenging problem with a wide variety of applications, since this control allows the design of acoustic or elastic lenses^1^,^2^,^3^,^4^,^5^,^6^, omnidirectional absorbers^7^,^8^,^9^, cloaking devices^10^,^11^,^12^ or hyperlenses^13^. The propagation of mechanical waves can be tailored by means of inhomogeneous materials, as it is well known that in these media waves follow curved trajectories that can be properly designed if the position-dependent parameters of the medium are chosen according to specific laws. However, it is obvious that natural materials cannot provide these specific position-dependent parameters. Therefore, their realization has to be achieved artificially.

In this context, the field of phononic crystals^14^,^15^ and metamaterials^16^ provides a promising background for the realization of artificial inhomogeneous materials, as has been widely demonstrated in the literature^17^,^18^,^19^,^20^,^21^,^22^.

The major drawback for the realization of these devices for elastic waves is that, unlike acoustic waves in fluids, the propagation of elastic waves, either in bulk materials or plates, presents three polarizations, which propagate at different speeds. Their design has been done so far for only one of these polarizations, which obviously hinders the full functionality of the devices for applications like cloaking, absorption or even energy harvesting.

The authors have recently developed a design method which allows the simultaneous control of two of the three fundamental plate modes^23^, namely the anti-symmetric (*A*_0_) and symmetric (*S*_0_) Lamb modes. This simultaneous control was based on an effective medium theory developed for the *A*_0_ mode^24^ working with Kirchhoff equation^25^, which is a two-dimensional equation describing the propagation of flexural waves in thin plates. The mentioned theory can be applied as well to the control of the *S*_0_ mode given that the refractive indexes of these two modes are related by means of the same elastic constants and the thickness of the plate, however the refractive index of the third plate mode, named the shear horizontal (*SH*_0_) mode, cannot be controlled by this approach, given that it depends on different elastic constants that, in general, cannot be changed independently. Due to the extraordinary difficulty required to work on homogenization using plate theories including the shear mode^26^, we propose in this work an alternate approach, based on the homogenization of the bulk phononic crystal.

In this work a homogenization theory for phononic crystals is developed and applied to the homogenization of phononic crystal plates, which provides the solution of the refractive indexes of the three modes. It is shown then that by means of a complex unit cell, consisting of a circular inclusion with a hole in its center, it is possible to design independently the refractive index of the three fundamental plate modes. The method is applied to the design of a flat gradient index lens, a Luneburg lens and a Maxwell lens working identically for the three modes. Finally, to illustrate the advantage of the method, a more advanced multi-lens device is designed, which works as a Luneburg lens for the *S*_0_ and the *SH*_0_ modes while it works as a Maxwell lens for the *A*_0_ mode. Numerical simulations by COMSOL support their functionality in a broadband frequency region.

## Fourier Homogenization of Phononic Crystals and Plates

Phononic crystals consist of periodic arrangements of inclusions in an elastic matrix. It is well known that for low frequencies these structures behave like homogeneous materials with some effective parameters (mass density ρ^*^ and stiffness tensor 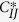). The well known Plane Wave Expansion method can be used to compute the dispersion relation of elastic waves in phononic crystals once we know the Fourier components of the position-dependent elastic constants, and the low frequency analysis of these equations can be used to obtain the effective elastic constants as


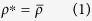



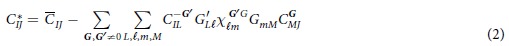


where 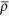 and 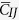 stands for the unit cell average of the mass density and stiffness tensor components, respectively, and ***G*** are the reciprocal lattice vectors of the periodic arrangement, and in all the expressions the term ***G*** = **0** is excluded. The quantities 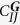 are the Fourier components of the position-dependent stiffness tensor components *C*_*IJ*_, for *I*, *J* = 1,…6, and the matrix components 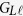 and *G*_*mM*_ are obtained from the components of the ***G*** vector in Voigt notation^27^. It must be pointed out that the above theory is valid for 1, 2 or 3-dimensional phononic crystals, being the dimensions of the ***G*** vector the only difference for each periodicity. Finally, the elements of the 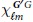 matrix are obtained as the inverse of the 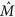 matrix defined as^27^


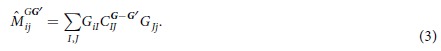


A phononic crystal plate is a two-dimensional periodic arrangement of inclusions in an elastic plate, and it has also been shown that for low frequencies these structures behave like homogeneous elastic plates. However, the analysis of the dispersion relations of these structures is more complex than that of their bulk counterparts, given that we require additional equations to satisfy boundary conditions, therefore the homogenization of these structures is a more complicated problem^28^. The authors have recently studied the focalization of the symmetric and antisymmetric Lamb modes in a phononic crystal plate^23^ assuming that, in the low frequency limit, this plate can be considered a “finite slice” of a two-dimensional phononic crystal, and an excellent agreement was found in the comparison of the respective dispersion curves. Therefore, it is possible to homogenize the bulk phononic crystal by means of equations (1) and (2), and analyze the vibrations of homogeneous plates with the given effective parameters. Although this study was done for the antisymmetric (*A*_0_) and symmetric (*S*_0_) Lamb modes, here its validity will be demonstrated for the three fundamental Lamb modes.

**Figure 1:**
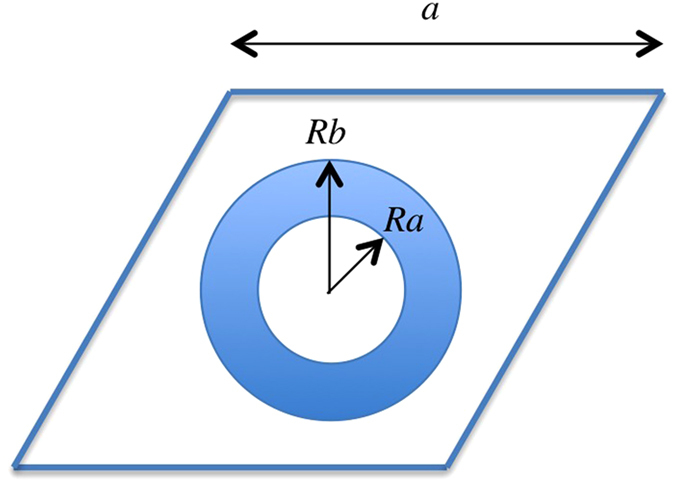
Unit cell employed in the design of GRIN devices, consisting of a triangular arrangement of gold inclusions of radius *R*_*b*_ with holes of radius *R*_*a*_ in an Aluminium matrix.

**Figure 2:**
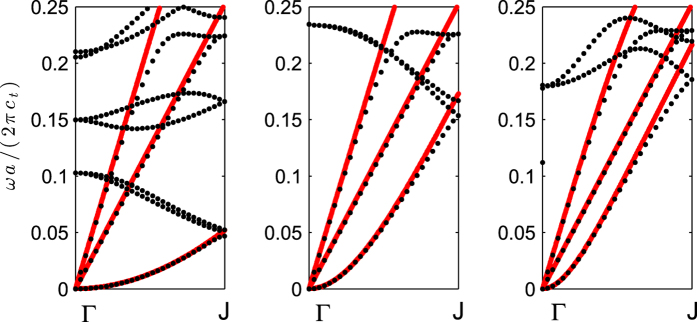
Dispersion relations for a phononic crystal consisting of a triangular lattice of gold shell - hole structure with inner radius *R*_*a*_ = 0.2*a* and outer radius *R*_*b*_ = 0.4*a* in an aluminum plate of thickness *h* = 0.1*a*, *h* = 0.5*a* and *h* = *a*, corresponding to the left, middle and right panels, respectively. Black dots show the curves calculated by COMSOL and red lines show the dispersion relations obtained by equation (4) with effective parameters in Table 1.

As an example, let us consider a triangular arrangement with lattice constant *a* of gold shell-hole structure, with inner radius *R*_*a*_ = 0.2*a* and outer radius *R*_*b*_ = 0.4*a*, in an Aluminum matrix (see elastic parameters in Table 1 and schematics of the unit cell in Fig. 1). In the low frequency limit this arrangement of inclusions behaves like a transversely isotropic solid whose effective parameters can be computed using equations (1) and (2) and are given in Table 1. If we take a plate of thickness *h* of such an effective solid, the dispersion relation of the three fundamental modes in the low frequency limit is isotropic and given by the following equations^23^


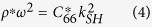



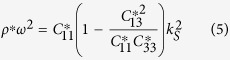



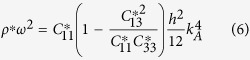


with *k*_*X*_ being the wavenumber of the *X* mode, for *X* = *SH*, *S*, *A*. Figure 2 shows the comparison of the full dispersion relation of the phononic crystal plate computed with COMSOL (black dots) with the dispersion relation obtained by means of equation (4) (red lines) for plate’s thickness *h* = 0.1*a*, 0.5*a* and *h* = *a* in left, center and right panels, respectively. It is clear how in the low frequency limit the plate can be described by the effective medium theory developed for bulk phononic crystals, which in turns simplifies considerably the calculations and also allows for the design of refractive devices, as will be shown in the following section.

## Simultaneous Control of the Fundamental Plate Modes

When a wave passes from a given medium I to a different one II it suffers refraction. The condition for refraction is derived by imposing conservation of the wavenumber in the direction parallel to the plane dividing the two media, and this condition defines the refractive index as the ratio between the wavenumber in the two media^29^. From expression (4) we find that the refractive indexes of the three fundamental modes are therefore given by


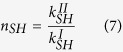



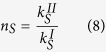



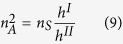


where it has been assumed that medium I(II) is a plate with elastic parameters labeled by I(II) and thickness *h*_*I*_ (*h*_*II*_). We can see therefore that the thickness of the plate can be used to control the refractive index of the *A*_0_ mode independently of the other two modes. Besides, we see that while the elastic constants defining the refractive index of the *A*_0_ and *S*_0_ mode are the same, the refractive index *SH*_0_ mode depends on the 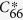 component of the stiffness tensor.

The design of gradient index devices is typically done by choosing one material for the background, another for the inclusions, and then the refractive index is a function of the filling fraction (radius) of the inclusions, that is, *n* = *n*(*R*_0_). In this work we have three refractive indexes to design, however the refractive index of the *A*_0_ mode will be designed by changing the relative thickness of the plate and using equation (9), so that actually we have to design only *n*_*S*_ and *n*_*SH*_. If we had only one degree of freedom, that is, the radius of the inclusion *R*_0_, we could choose for instance *n*_*S*_ and then determine the corresponding value of *R*_0_, since *n*_*S*_ = *n*_*S*_(*R*_0_), but then the refractive index of the *SH*_0_ mode would be imposed, so that we need an additional degree of freedom to choose independently *n*_*S*_ and *n*_*SH*_.

We propose the structure shown in Fig. 1, in which an Aluminum matrix is perforated by a triangular arrangement of gold inclusions of radius *R*_*b*_ with a hole at its center of radius *R*_*a*_ (see materials’ parameters in Table 1). The effective parameters of such a phononic crystal will therefore be a function of both *R*_*b*_ and *R*_*a*_, which will allow us, as will be explained later, the independent tuning of the three refractive indexes.

**Figure 3:**
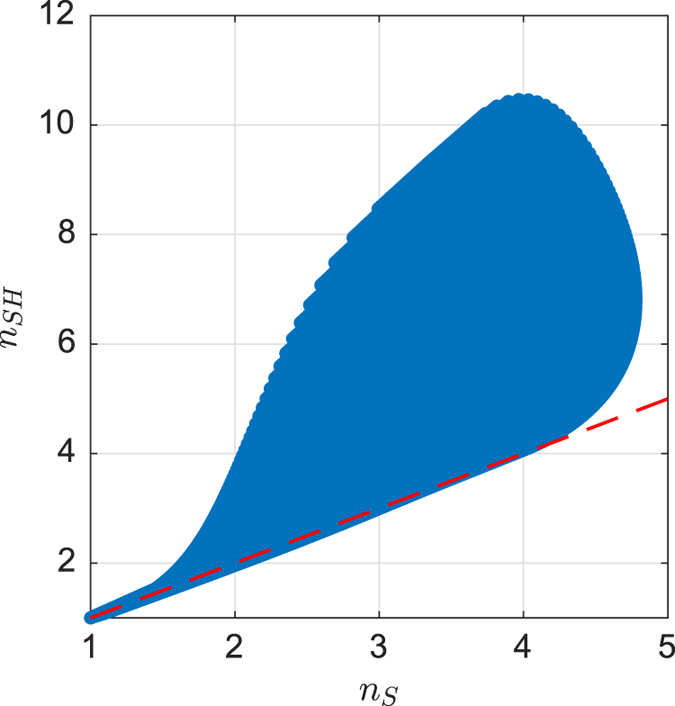
Phase diagram showing all the possible values for the effective refractive indexes for the *SH*_0_ and *S*_0_ modes obtained by varying the radius of the holes and the inclusions. The red-dashed line shows the condition *n*_*SH*_ = *n*_*S*_, showing that it is possible the design of refractive devices identical for these two modes.

Figure 3 shows the “phase diagram” of this material, in which we make a sweep of *R*_*b*_ ∈ (0, 0.5*a*) and *R*_*a*_ ∈ (0, *R*_*b*_), with *a* being the lattice constant of the arrangement. All the possible values that take *n*_*S*_ and *n*_*SH*_ are shown in this diagram. This diagram can be used as an inverse design tool: We choose a given value for the desired refractive index for the two modes lying in the diagram, then we find the corresponding values for *R*_*a*_ and *R*_*b*_, to later use expression (9) to determine the thickness of the plate that give us the desired value for *n*_*A*_, so that we are capable of independently tune the three refractive indexes, obviously under the limitations given by the phase diagram depicted in Fig. 3.

## Numerical Examples

This section is devoted to illustrating the power of the presented method to design gradient index devices working simultaneously for all three fundamental plate modes. The design procedure is identical for all these devices: First we decide the device, defined by means of a position-dependent refractive index; second, we define the positions of a cluster of inclusions, according to the device’s geometry (a square slab for a flat GRIN and a circular cluster for a Luneburg or Maxwell lenses), with a large enough number of inclusions to avoid diffractive effects (see discussion below); third, we select the inclusion *α* and compute the desired radii *R*_*a*_ and *R*_*b*_. For the computation of these values we need to know the desired refractive indexes for the *S*_0_ and *SH*_0_ modes at the position ***r***_*α*_, and then use the data of the phase diagram shown in Fig. 3 which relates the refractive index with the radius of the hole and inclusion. Finally, once we have selected *R*_*a*_ and *R*_*b*_, by means of equation (9) we choose the thickness of the plate at that position to obtain the desired refractive index for the *A*_0_ mode. It has to be noticed that this design method allows for creating refractive devices for the three modes, but not necessarily the same device for the three modes, given that it can be used to tune independently the three indexes. Therefore, although from the practical point of view it appears better to design devices focusing all the energy at the same point for one kind of lens, we propose in our last example the realization of a multi-lens device working as a Luneburg lens for the *S*_0_ and *SH*_0_ modes and as a Maxwell lens for the *A*_0_ mode.

**Figure 4:**
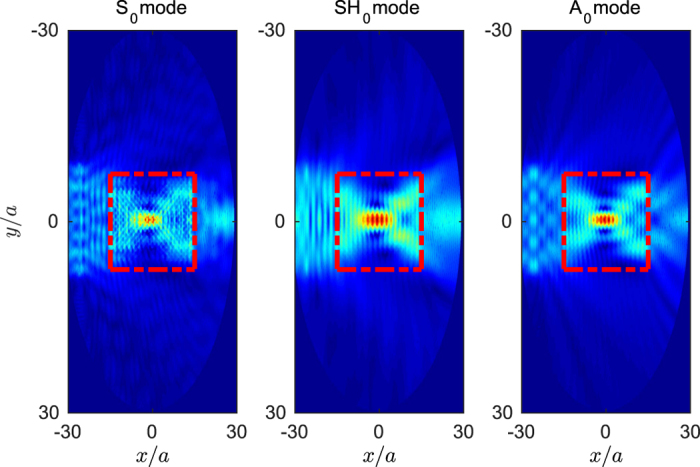
GRIN flat lens made with a slab of 15 rows and 30 columns of gold-hole inclusions in an Aluminum plate of thickness *h* = *a*. Simulations are shown at the same frequency *ωa*/2*πc*_*t*_ = 0.178, which corresponds to wavenumbers *k*_*S*_*a* = 0.65, *k*_*SH*_*a* = 1.11 and *k*_*A*_*a* = 1.5, whose corresponding field distributions are shown in left, central and right panels.

Figure 4 shows a COMSOL simulation of the first example considered in this work. It consists in a long GRIN flat lens as described in several works^1^,^23^, in which the refractive index is a function of the distance *y* to the center of the lens and it is given by


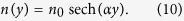


In this specific case, the lens is made of 15 rows and 34 columns of inclusions arranged in a triangular lattice, oriented in such a way that the vertical distance between inclusions equals the lattice constant *a*, therefore it has a height *L*_*y*_ = 15*a* and a width 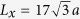. The plate has a thickness *h* = *a* in the background (recall that the device has a position-dependent thickness). The simulations show the total displacement field when an external plane wave of a given polarization propagates through the *x* axis and it arrives to the device. Results shown in the left, central and right panels correspond to the *S*_0_, *SH*_0_ and *A*_0_ modes, respectively, and they are computed at the same frequency *ωa*/2*πc*_*t*_ = 0.178, which corresponds to wavenumbers *k*_*S*_*a* = 0.65, *k*_*SH*_*a* = 1.11 and *k*_*A*_*a* = 1.5. The dotted red lines display the size of GRIN flat lens, where the focusing points are demonstrated near the center as expected for all three modes.

We can see how the quality of the focusing point depends on the wavenumber of the incident field, given that it will be limited by diffraction and size effects. Effectively, it is commonly assumed in phononic crystals based devices that the limit of validity is the “low frequency limit”, however in those situations in which we have more than one mode propagating through the material, this limit has to be properly analyzed. The homogenization limit is indeed a propagation regime in which the wavelength of the propagating field is larger than the typical distance between scatterers, so that the field actually detects an average medium and cannot distinguish individual scatterers. Therefore, it is the wavelength of the field and not its frequency the relevant quantity, for this reason, for the same frequency, the different modes will have different wavelengths, and therefore different responses.

In general, there are two physical phenomena relating the validity of the functionality of the device. The upper limit in wavelength is limited by the fact that this wavelength should be smaller than the size of the device, so that the refraction effect dominates over diffraction, this limit can obviously be controlled by increasing the size of the device and, therefore, the number of inclusions; The lower limit in wavelength is determined by the validity of the homogenization theory, which requires a wavelength typically larger than at least 3 or 4 times the lattice constant. These limits are not strictly defined and are only approximate, however it is obvious that for the same device they will be at different frequencies for the three modes, although it is clear that there will be a frequency region in which these conditions hold for the three modes.

The second example considered in this work is a Luneburg lens, which consists of a circular lens in which the refractive index depends on the position to the center of the lens as^30^


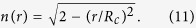


**Figure 5:**
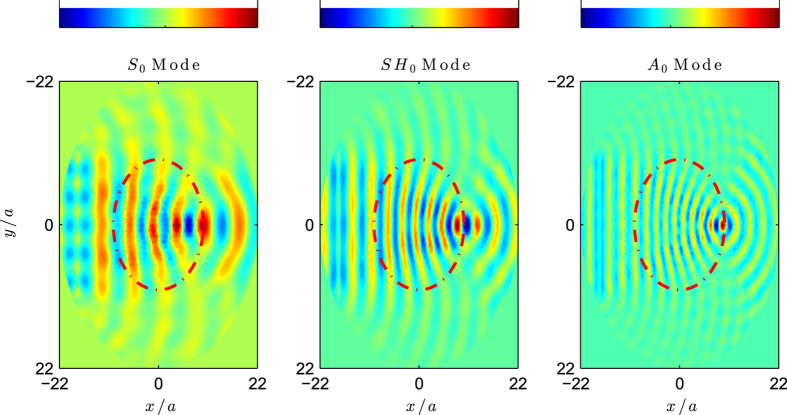
Luneburg lens of radius *Rc* = 10*a* with about 300 inclusions. Simulations are shown at *ωa*/2*πc*_*t*_ = 0.24, which corresponds to wavenumbers *k*_*S*_*a* = 0.87, *k*_*SH*_*a* = 1.5, and *k*_*A*_*a* = 1.74, whose corresponding field distributions are shown in left, central and right panels.

This refractive index is designed in such a way that any plane wave arriving to the lens is focused at the diametrically opposed border of it. It is therefore an omnidirectional device, as it is isotropic and radially symmetric. Its realization is identical to that of the GRIN lens, but this time we use a circular cluster of inclusions of radius *R*_*c*_ = 10*a*. Figure 5 shows a COMSOL simulation in which a plane wave of a given polarization impinges the cluster from the left, and it is therefore focused at the opposite side of the cluster. The simulations show the real part of the component of the displacement field dominant for each polarization (*u*_*x*_ for the *S*_0_ mode, *u*_*y*_ for the *SH*_0_ mode and *u*_*z*_ for the *A*_0_ mode) and they are performed as in the previous example at the same frequency *ωa*/2*πc*_*t*_ = 0.24, corresponding to *k*_*S*_*a* = 0.87, *k*_*SH*_*a* = 1.5, and *k*_*A*_*a* = 1.74, shown in left, central and right panels, respectively. From the simulations it is clear the path followed by the wavefront which is finally focused at the border of the lens, as expected.

The following example is similar to the previous one, but this time the proposed omnidirectional device is a Maxwell lens^30^, whose refractive index is given by


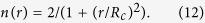


**Figure 6:**
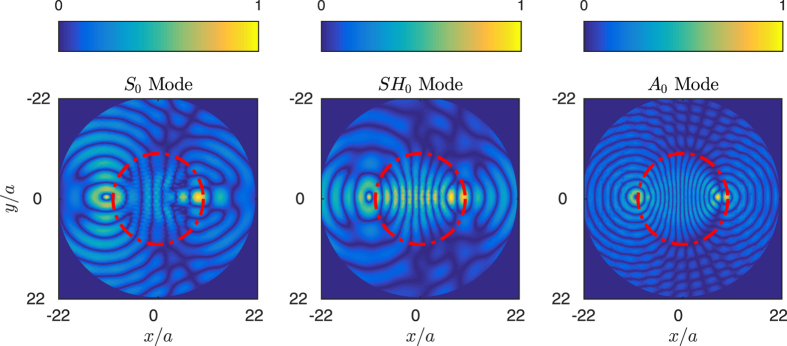
Maxwell lens of radius *Rc* = 10*a* with about 300 inclusions. Simulations are shown at *ωa*/2*πc*_*t*_ = 0.178, which corresponds to wavenumbers *k*_*S*_*a* = 0.65, *k*_*SH*_*a* = 1.11, and *k*_*A*_*a* = 1.5, whose corresponding field distributions are shown in left, central and right panels.

The Maxwell lens is designed in a way that a point source excited at one border of the lens is focused at the opposite side of it. The design method is the same as for the Luneburg lens, and Fig. 6 shows the real part of the results of the simulations performed with COMSOL. The point source for each polarization is excited as a body force in the *x*, *y* and *z* direction for the *S*_0_, *SH*_0_ and *A*_0_ modes, respectively.

As before, the plots show the real part of the dominant component of the displacement field for each polarization. The source is excited at *x* = −10*a* at the same frequency *ωa*/2*πc*_*t*_ = 0.178, corresponding to *k*_*S*_*a* = 0.65, *k*_*SH*_*a* = 1.11 and *k*_*A*_*a* = 1.50, shown in left, central and right panels, respectively. The focusing points are identically located at the *x* = 10*a* border for the three modes, which shows the good performance of the device.

The above simulations show the performance of the method for the simultaneous control of the three fundamental modes, although the three devices work identically for the three modes. However, the method does not require that the refractive index profile be the same for the three modes, since each refractive index can be tuned independently, which means that a device can be designed as working as one type of lens for one mode and as different one for another mode.

**Figure 7:**
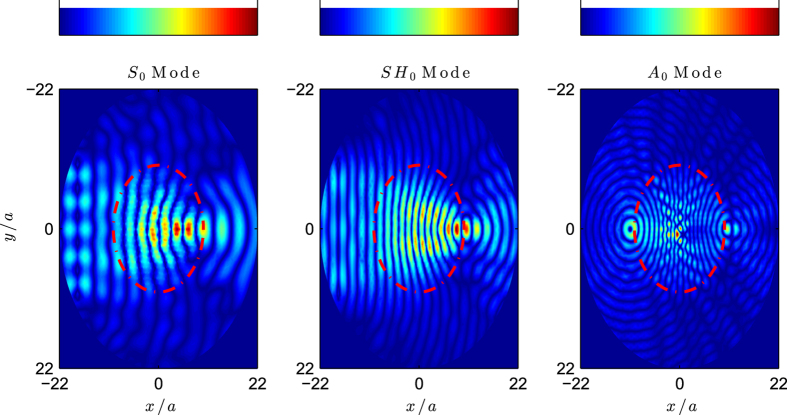
Luneburg and Maxwell lens working at the same wavenumber for the three modes *ka* = 1.5 which corresponds to the frequencies *ωa*/*c*_*t*_ = 0.41, 0.24 and 0.17 for the *S*_0_, *SH*_0_ and *A*_0_ polarizations, respectively.

To demonstrate the power of the present method, a multi-lens device is designed to behave as a Luneburg lens for the *S*_0_ and *SH*_0_ modes and as a Maxwell lens for the *A*_0_ mode. Figure 7 shows the real part of the dominant component of the displacement field for each polarization, but this time the simulations were made at the same wavenumber *ka* = 1.5, in this way it is easier to compare the field distributions and performance of the device. This wavenumber corresponds to reduced frequencies *ωa*/*c*_*t*_ of 0.41, 0.24 and 0.17 for the *S*_0_, *SH*_0_ and *A*_0_ modes respectively. We see how the device behaves as a Luneburg lens for the *S*_0_ and *SH*_0_ modes while it behaves as a Maxwell lens for the *A*_0_ polarization.

## Summary

In summary, we have presented a method for the design of refractive devices working simultaneously for the three fundamental Lamb modes in thin plates. The method is based on the homogenization of phononic crystal plates, studied here as finite slices of phononic crystals. A complex unit cell is employed to simultaneously control the refractive index of the three modes, together with thickness variations of the plate, the system has therefore enough degrees of freedom to independently tune the refractive indexes of the three modes.

The performance of the method is demonstrated by means of the design of a flat gradient index (GRIN) lens and a circular Luneburg and Maxwell lens working simultaneously for the three modes. Also, a more advanced device is shown which consists in a circular lens working as a Luneburg lens for the *S*_0_ and *SH*_0_ polarizations and as a Maxwell lens for the *A*_0_ one.

Given that the design method is based on a homogenization theory in the quasi-static limit, the devices are shown to work in a broad frequency region, so that the presented method can be efficiently employed to the design of devices for the control or harvesting of mechanical energy, since it allows the full control of vibrations excited in a finite elastic plate. Finally, it must be pointed out that this method can be applied to more complex waves in solids or fluids, being therefore a general approach for the control of multimodal mechanical waves.

## Additional Information

**How to cite this article**: Jin, Y. *et al.* Gradient Index Devices for the Full Control of Elastic Waves in Plates. *Sci. Rep.* **6**, 24437; doi: 10.1038/srep24437 (2016).

## Figures and Tables

**Table 1:** Elastic parameters of the materials used in the text.

Material	*ρ* (Kg/m^3^)	*C*_11_ (GPa)	*C*_12_ (GPa)	*C*_33_ (GPa)	*C*_13_ (GPa)	*C*_66_ (GPa)
Aluminum	2.7E3	108.2	51.2	*C*_11_	*C*_12_	(*C*_11_ − *C*_12_)/2
Gold	19.5E3	190	161	*C*_11_	*C*_12_	(*C*_11_ − *C*_12_)/2
Effective Material	9.63E3	61.1	32.3	79.2	36.6	(*C*_11_ − *C*_12_)/2
